# Neuromuscular block monitoring by smartphone application (i-TOF^©^ system): an observational pilot study

**DOI:** 10.1038/s41746-020-00344-w

**Published:** 2020-10-20

**Authors:** Lorenzo Schiavoni, Giuseppe Pascarella, Stefania Grande, Felice Eugenio Agrò

**Affiliations:** Unit of Anaesthesia, Intensive Care and Pain Management, Department of Medicine, Università Campus Bio-Medico di Roma, Via Álvaro del Portillo 21, 00128 Rome, Italy

**Keywords:** Nerve conduction studies, Information technology

## Abstract

Neuromuscular block monitoring is recommended by international guidelines to improve myorelaxation during surgery and reduce the risk of postoperative residual curarization. We conducted a pilot study to verify the efficacy of i-TOF, a wireless neuromuscular monitoring device connectable to a smartphone, comparing it with TOF WATCH SX. We enrolled 53 patients who underwent general anesthesia. For each patient, we recorded by both devices, in different time intervals, train-of-four (TOF) count/ratio after induction to general anesthesia (TI0–TI3) and during recovery (TR0–TR3). Moreover, post-tetanic count (PTC) was evaluated during deep neuromuscular block (TP0–TP2). We noticed no significant differences between the devices in recorded mean values of TOF ratio, TOF count, and PTC analyzed at time intervals for every phase of general anesthesia, although the i-TOF tends to an underestimation compared to TOF WATCH SX. For each patient, data sessions were successfully recorded by a smartphone. This aspect could be relevant for clinicians in order to have a stored proof of good clinical practice to be added on anesthesiologist records. By our results, i-TOF demonstrates a comparable efficacy to TOF WATCH SX, suggesting that it could be a proven alternative to standard devices for neuromuscular block monitoring. Further studies are needed to confirm our findings.

## Introduction

Neuromuscular blockade has a fundamental role during general anesthesia, together with analgesia and hypnosis, involving both anesthesiologic and surgical aspects^1^.

Paralysis induced by neuromuscular blocking agents ensures optimal conditions for managing airways and improves the quality of surgical field^2,3^. Moreover, deep neuromuscular blockade demonstrates to reduce postoperative pain in several surgical settings^4^. Nowadays, neuromuscular block (NMB) monitoring during general anesthesia is strongly recommended by international guidelines^5–7^. The reasons include avoiding postoperative residual curarization (PORC), maintaining optimal surgical conditions, and decreasing postoperative morbidity and length of stay^4,8^.

Neuromuscular monitoring devices are based on three different kinds of quantitative analysis: acceleromyography, electromyography, and mechanomiography^9^. Among these, acceleromyography is the most used technique in daily clinical practice, measuring the acceleration of a muscle movement, due to the neurostimulation of a specific nerve, i.e., ulnar, anterior tibial, or orbicular branch of facial nerve^10^. Acceleromyography continuously records, analyzes, and displays important parameters, such as a single twitch (ST) and train of four (TOF), making it usable for the clinicians and facilitating a rational approach to administration of neuromuscular blocking agents and their antagonists^8^.

Actually, we have two different kinds of neuromuscular monitoring devices, based on accelerometric analysis: integrated and “stand alone” devices. While the former requires a specific dedicated working station and monitors, the latter are portable, small, and simple to use^11^.

**Fig. 1:**
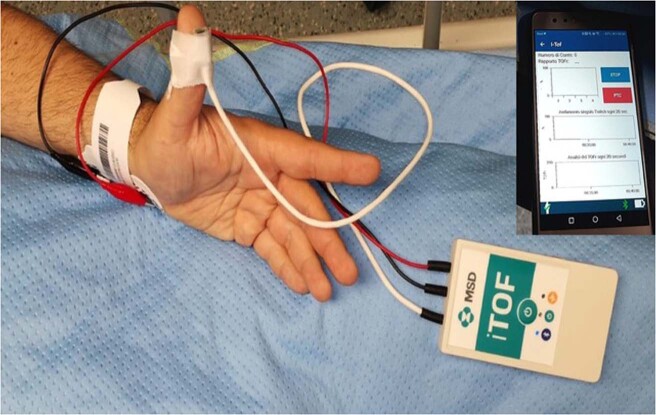
The i-TOF Device. The i-TOF device consists of a triaxial accelerometer, two poles for nerve stimulation, and wireless connection on a smartphone by application.

We conducted a pilot study to verify the efficacy of i-TOF (Vree Health, Italy & MSD, USA), a new wireless neuromuscular monitoring device that integrates Bluetooth technology combined with an application downloadable on a smartphone (Fig. 1). Besides the absence of connection wires, the advantages of this new device include portability, since the personal smartphone can work as a remote display, together with the possibility to save measured data, which can be attached to the patient’s medical record or used for clinical study purpose.

We compared i-TOF with the more studied and validated TOF WATCH SX (Organon, Ireland)^12–15^, using both simultaneously on 51 patients undergoing general anesthesia.

The aim of our study was to demonstrate an equivalent efficacy in measurement of the following neuromuscular parameters: ST, TOF count, TOF ratio, and post-tetanic count (PTC). As a secondary outcome, we verify the absence of any adverse effect using i-TOF.

## Results

### Neuromuscular parameters

Fifty-three patients were included in the study. The patient’s mean age was 51.6 ± 14.4 years. Mean total duration of anesthesia was 92 ± 68.3 min. The types of surgical procedures are listed in Table 1. Neurostimulation was performed on ulnar nerve in 50 patients, while on tibial nerve in three patients. Mean dose of rocuronium was 49.3 ± 14.4 mg.

**Table 1:** Number of patients undergoing different surgical procedures.

Surgical procedure	No.	Surgical procedure	No.
Functional endoscopic sinus surgery	15	Cholecystectomy	1
Duct and sac drainage	5	Hysterectomy	1
Vestibular neurectomy	1	Appendicectomy	1
Acoustic neuroma excision	1	Tonsillectomy	1
Sleeve gastrectomy	5	Mammaplasty	1
Rhinoplasty	4	Microlaryngoscopy	3
Pharyngoplasty	2	Abdominal aortic aneurysm resection	1
Cystectomy	1	Abdominal wall plasty	2
Colpohysterectomy	1	Interaorto-caval limphadenectomy	1
Tympanoplasty	2	Diagnostic laparoscopy	1
Nasal polipectomy	1	Gastric bypass	2

**Fig. 2:**
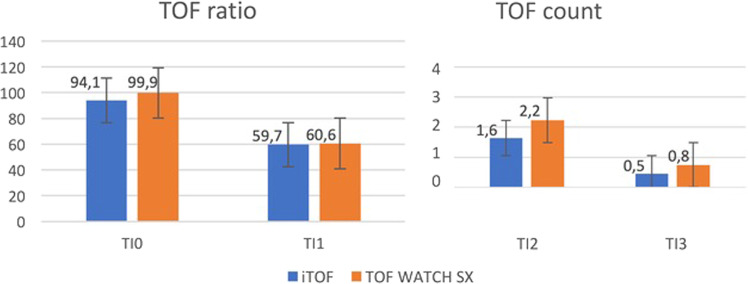
Neuromuscular parameters during the induction to general anesthesia. Diagram comparing means of the TOF ratio (left side, expressed in percentage) and TOF count (right side, expressed in absolute value) between i-TOF and TOF WATCH SX among the different induction times.

Regarding the induction phase, we mainly report no significant differences in medium TOF values between i-TOF and TOF WATCH SX, respectively, a TOF ratio of 94.1 ± 31.3% vs. 99.9 ± 16% (*p* = 0.09) at TI0, and 59.7 ± 27.8% vs. 60.6 ± 35.8% at TI1 (*p* = 0.41), while a TOF count of 1.6 ± 1.5 vs. 2.2 ± 1.5 (*p* < 0.05) at TI2, 0.5 ± 0.93 vs. 0.8 ± 1.11 (*p* = 0.08) at TI3 (Fig. 2).

**Fig. 3:**
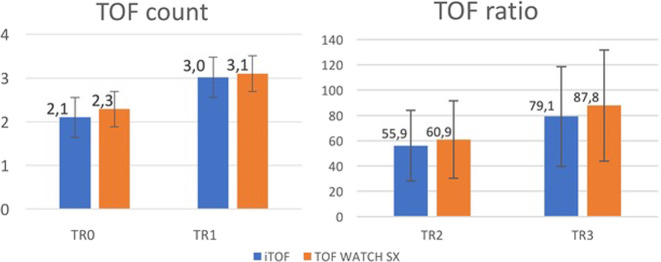
Neuromuscular scores during the recovery period. Diagram comparing means of the TOF count (left side, expressed in absolute value) and TOF ratio (right side, expressed in percentage) between i-TOF and TOF WATCH SX among the different recovery times.

Tests during the recovery phase demonstrate that there was no statistical difference between the two devices, showing a TOF count of 2.1 ± 1.5 vs. 2.3 ± 1.7 (*p* = 0.15) at TR0, 3 ± 1.4 vs. 3.1 ± 1.3 (*p* = 0.55) at TR1, while a TOF ratio of 55.9 ± 34.8% vs. 60.9 ± 59.7% (*p* = 0.28) at TR2, 79.1 ± 33.9% vs. 87.8 ± 53.4% (*p* = 0.14) at TR3 (Fig. 3).

**Fig. 4:**
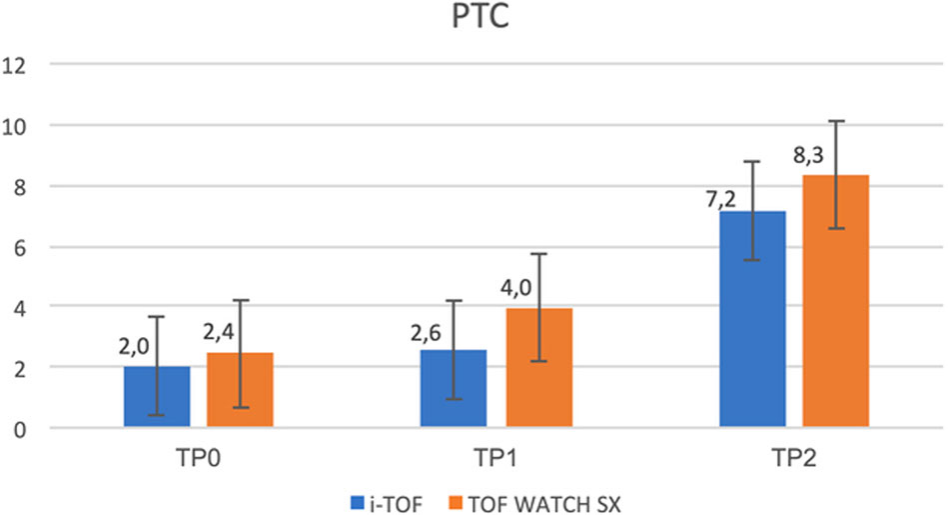
Neuromuscular parameters during a deep neuromuscular blockade. Diagram showing the progressive rise of PTC, recorded during deep NMB, between i-TOF and TOF WATCH SX among the different times.

At last, we demonstrated no difference in monitoring PTC during deep neuromuscular blockade (2 ± 3.7 vs. 2.4 ± 3.7 at TP0, *p* = 0.27; 2.6 ± 3.4 vs. 4 ± 5.1 at TP1, *p* = 0.06; 7.2 ± 6.7 vs. 8.3 ± 7.1, *p* = 0.11 at TP2) (Fig. 4).

### Secondary outcomes

**Fig. 5:**
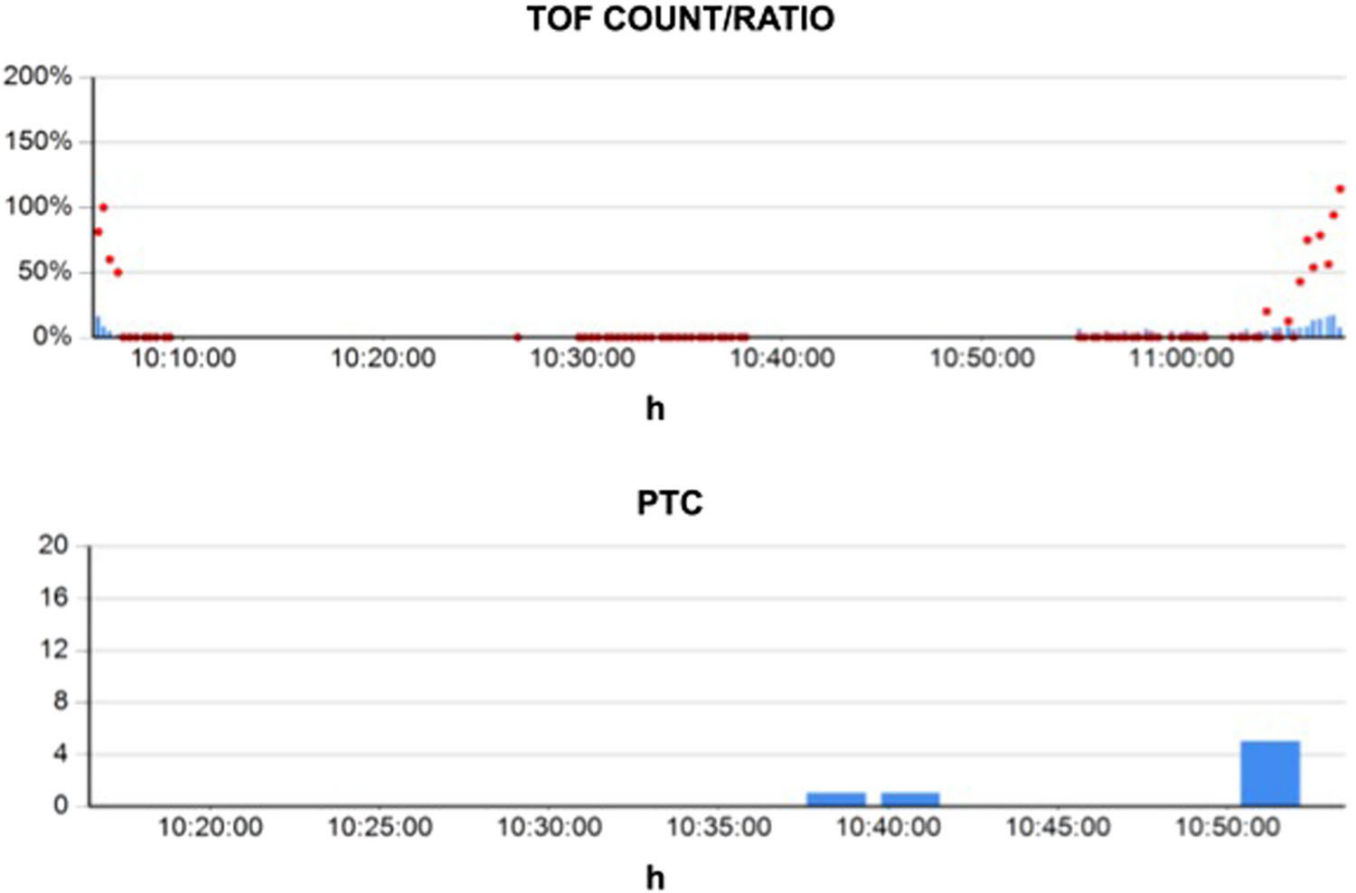
The i-TOF report. The i-TOF report: an example of a data report created by the I-TOF application on a smartphone during a session. The upper side shows values of TOF count (blue columns) and TOF ratio (red dots). The lower side shows values of PTC; h hours.

For each session, the application successfully produced a report, showing the timeline of TOF count/ratio and PTC in two different overlapping diagrams, clearly displaying the transition from complete neuromuscular activity to moderate neuromuscular blockade and then to deep neuromuscular blockade, and finally came back to recovery throughout the inverse sequence (Fig. 5).

Moreover, other events could be manually recorded through the application over the graph, such as NMB or reversal administration by the clinician. The report can be easily sent to an e-mail address or saved in the memory of the smartphone device. No adverse effect regarding the use of i-TOF was present. Furthermore, on the basis of correct information provided by both devices, we did not detect any case of PORC, neither uncomplete relaxation of vocal cords during tracheal intubation.

Regarding the secondary endpoints, disconnections have shown to be frequent and in most cases determined by i-TOF battery-saving modality with an amount to 2,8 disconnection for each case. We verified a total of 8 disconnections with loss of data and 15% of reports failed to be saved.

Battery life was improved by energy-saving mode due to the new Bluetooth protocol and forced standby after 10 min of disuse. However, a single battery (composed of one 9-V battery) revealed a duration of approximately 12 h, enough for a daily surgery session.

Connection was always very fast during sessions and covered a maximum distance of 3 m between a smartphone and i-TOF device.

## Discussion

The monitoring of neuromuscular blockade has been stated as a reference standard during general anesthesia by international recommendations^5–7^. However, the use of neuromuscular monitoring devices is not yet a consolidated standard in clinical routine^16,17^, and this condition increases the risk of postoperative morbidity due to the residual effects of neuromuscular blocking drugs^18^.

But why are clinicians so negligent in using neuromuscular monitoring?

Some reasons have been discussed: cost of the devices, the lack of adequate training, and even a problem of hubris, as maybe many anesthesiologists think that they can rely on clinical signs^19^.

Thus, at the current time, it appears that one of the main barriers to routine adoption of quantitative monitoring is the lack of availability of an easy-to-use monitor^11^. Many devices for neuromuscular monitoring are available on the market: it is probably only a matter of simplifying their usage in daily clinical life. We live in a world in which most of our daily activities are managed by smartphone applications, and this allows us to manage them quickly and easily: so why not apply the same discourse to our clinical activity?

For such reasons, we conducted an observational study to assess the efficacy of a new neuromuscular monitoring device i-TOF consisting of an accelerometer connected wirelessly to the smartphone by an application, where we could visualize the data recorded. The application is well designed, smart, and intuitive: this was confirmed by all the operators involved in the study, as no difficulty was recorded about usage. We compared i-TOF to another stand-alone device, the TOF WATCH SX, whose efficacy has already been shown by several works^12–15^.

In our study, we mainly found an overlap between the two devices in recorded mean values of TOF ratio, TOF count, and PTC analyzed at time intervals for every phase of general anesthesia, although the i-TOF tends to an underestimation compared to TOF WATCH SX.

The only significant difference was found in TI2; this may probably be due to the different calibration systems of the devices (i-TOF is equipped with an automatic system). However, this difference is not relevant to clinical decisions, as both the mean values of TOF count among the groups in TI2 are ≥1, which means a moderate block, not indicated for tracheal intubation.

PTC values showed a high variability, expressed in high standard deviations, but it regards both the devices and falls in a value range that does not modify the clinical conduit, as any value of PTC ≥ 1, which means a deep block does not impact on dose administration of NMB agents rather than reversal. The best match between i-TOF and TOF WATCH SX was found in the recovery phase, which has the most clinical relevance, as an effective neuromuscular monitoring guides the anesthesiologist in the correct administration of reversal drugs, preventing PORC.

Nevertheless, neuromuscular monitoring devices have some limits: when handling the display, an accidental disconnection from the wires can happen or a displacement of the electrodes due to a traction by the wires themselves. Other limits include the requirement for baseline measurements, the reduced precision in awake patients, and the lack of storage for data recorded^20–22^. The latter aspect could be relevant for clinicians in order to have a stored proof of good clinical practice to be added on anesthesiologist records.

Moreover, Brull et al. described the ideal features which a neuromuscular monitoring should have, including portability, connectivity, and presence of data memory^11^, and i-TOF has all these characteristics, including the wireless mode. We performed most of neurostimulation on the ulnar nerve because it is the preferred site of monitoring neuromuscular function^8^.

In three patients, we performed neurostimulation on the tibial nerve, which is indicated as an alternative to ulnar nerve^8^, because the hands were not accessible.

A limit of i-TOF could be represented by the first coupling with a smartphone, which requires some minutes to authorize connection, but every reconnection is fast and effective. Furthermore, the smartphone can be connected only with one device at the same time.

By our results, i-TOF demonstrates a comparable efficacy to TOF WATCH SX, suggesting that it could be a proven alternative to standard device for neuromuscular block monitoring.

Moreover, the possibility to manage neuromuscular monitoring and save the report sessions all by a smartphone could be an incentive to increase neuromuscular blockade monitoring in anesthesiologist practice. More studies are needed to confirm our findings.

## Methods

### Study design

We collected data on i-TOF comparing the results with TOF WATCH SX since induction of anesthesia to awakening. The i-TOF device consists of a box containing a triaxial accelerometer and two poles for nerve stimulation. A power button is located on the box with three leds indicating the status of battery, connection, and Bluetooth activity (Fig. 1).

The i-TOF connects with personal smartphones by an application released by manufacturers on online stores (Apple and Play store), compatible with both iOS and Android operative systems. The application access requires an alphanumeric password, then the device can be activated remotely from the application to start measurements. Furthermore, the i-TOF application is equipped with a firewall blocking all the incoming and outgoing connections, including connection attempts from other applications on the smartphone, except for the i-TOF device.

Since the introduction of i-TOF in our hospital, all the anesthesiologists received a single 30-min training about the use of this device. TOF WATCH SX was already used for several years in our department. Data were recorded by clinicians who were not necessarily study investigators.

After approval of our Ethical Committee (Prot. 18.18 TS ComEt), we recruited patients undergoing general anesthesia at the University Hospital Campus Biomedico of Rome from June 2018 to September 2019.

Eligibility criteria included signature of informed consent, age > 18, and ASA physical status ≤ 3.

Patients presenting neuromuscular morbidities or allergy to the NMB agent and reversal drug used in this study were excluded. Each patient was monitored in the operating room with both devices symmetrically, before the induction of anesthesia, choosing the best site to place the poles for neurostimulation in consideration of the type of surgery.

During deep sedation, before the injection of the NMB agent, both the devices were calibrated and then the first TOF ratio was recorded (TI0). Subsequently, from the injection of rocuronium 0.6 mg/kg, a TOF ratio/count was recorded at 1, 2, and 3 min (TI1–TI3).

At TI3, if the TOF count was less than 1, we proceeded with tracheal intubation, and maintenance with halogenated vapor or hypnotic agents in TCI was started. Ten minutes after induction to general anesthesia, we started measuring PTC, repeating it every 10 min for a total of three records (TP0–TP2). Management of curarization during maintenance of general anesthesia was left to clinicians’ judgment in relation to neuromuscular monitoring data and surgical needs.

In proximity of awakening, the anesthesist started TOF count/ratio detecting at TR0 (time for recovery), TR1, TR2, and TR3, each performed after 1 min from the previous one, and giving the reversal drug (sugammadex) as necessary. Every measurement was performed simultaneously by both devices.

Moreover, during each session, the following data regarding i-TOF were recorded:

- Number of disconnections with loss of session data on the application
- Duration of battery life previewed and effective
- Extension of Bluetooth^®^ range
- Speed of connection
- Efficacy in producing the report and saving data
- Presence of any adverse event related to i-TOF usage or to presented data

### Statistical analysis

Continuous data are presented as mean ± standard deviation, while categorical data are presented as absolute values (numbers and percentages).

*T*-student test has been used to compare continuous parametric data among different groups. All analyses and graphs were performed using the software Excel (Microsoft, USA). A *p* value of less than 0.05 was considered statistically significant.

### Reporting summary

Further information on research design is available in the Nature Research Reporting Summary linked to this article.

## Supplementary information

Reporting Summary

## Data Availability

The datasets generated and analyzed during the current study are available in the “Zenodo” public repository https://zenodo.org/record/3783621#.Xq7qMC-uZPM.
